# Recent Evidence on Factors and Potential Interventions Affecting Patient Sleep in Inpatient and Hospital Settings

**DOI:** 10.1007/s11920-026-01720-1

**Published:** 2026-10-09

**Authors:** John Nordstrand, Natalia Vincens, Michael G. Smith

**Affiliations:** 1 School of Public Health and Community Medicine, Institute of Medicine, Sahlgrenska Academy, University of Gothenburg, Gothenburg, Sweden; 2 Occupational and Environmental Medicine, University of Gothenburg, Box 453, Gothenburg, 405 30 Sweden

**Keywords:** Inpatient sleep, Hospital environment, Sleep disruption, Intensive care unit, Delirium, Non-pharmacological interventions

## Abstract

**Purpose of review:**

Sleep is a critical but frequently disrupted part of hospital care, affecting patient recovery, length of stay, and physical and mental health. This review examines recent evidence (2022–2026) on why inpatient sleep is disrupted and how interventions can mitigate disturbance across different patient groups and hospital settings.

**Recent findings:**

Findings span five domains: environmental factors such as noise and light, hospital infrastructure and room configuration, patient-related factors including pain and anxiety, care routines, and interventions. Recent high-quality trials and meta-analyses increasingly support nonpharmacological strategies - including environmental modification, care-routine restructuring, and sensory therapies - while evidence for pharmacological agents such as melatonin remains weak outside specific contexts.

**Summary:**

This review highlights the multifactorial nature of sleep disruption in hospital settings. Prioritizing patient sleep through evidence-based, sustainable strategies can support faster recovery, reduce complications, and improve overall care quality, yet poor sleep remains pervasive. Current evidence supports system-level interventions (workflow restructuring, environmental redesign) and low-cost measures (earplugs, sleep masks, physical therapy) to improve sleep related outcomes including delirium, pain, and length of stay.

## Introduction

Sleep of sufficient quantity and quality is a critical component of recovery for hospitalized patients. Adequate sleep supports surgical healing, improves immune function, and plays a vital role in preventing delirium, improving mood, and reducing anxiety during hospital stays [1, 2]. Despite its importance, many inpatients report significant sleep disturbances, which have been associated not only with poorer clinical outcomes, but also with prolonged hospital stays, and increased healthcare costs [3].

Sleep disturbances in hospital settings arise from multiple environmental, clinical, and organizational factors. Well-documented factors include frequent nighttime vital sign checks, elevated noise levels, and continuous monitoring, especially in intensive care units (ICUs) [4]. Sleep quality has been shown to vary across different hospital settings, with patients in ICUs often reporting the poorest sleep [5]. This may be due to, in addition to illness severity, exposure to artificial light, frequent staff interruptions, and shared accommodations [6, 7]. Accordingly, pharmacological and nonpharmacological interventions aimed at mitigating these factors - including eye masks, earplugs, staff education, care routine adjustment, relaxation therapies, and pain management - have been shown to improve sleep quality and clinical outcomes [1, 8, 9].

With growing recognition that poor inpatient sleep contributes directly to adverse clinical endpoints, recent years have seen more high-quality trials, better design of interventions, evolving outcomes of interest, expanding focus beyond only ICUs, and hospitals enacting policies to prioritize patient sleep. We aimed to synthesize key findings and research directions across studies on sleep in hospitals, examine interventions focused on inpatient sleep, and discuss their implications for clinical outcomes linked to sleep quality, including length of stay prolonged by sleep-related delays in healing, and elevated delirium risk through circadian disruption and neuroinflammation [2, 7]. We organize the evidence into five domains: environmental factors, hospital infrastructure and room configuration, patient-related factors, care routines, and interventions aimed at improving inpatient sleep.

## Methods

We performed a narrative review of recent (2022–2026) literature on factors influencing inpatient sleep across general hospital, intensive care, paediatric, and psychiatric settings. Relevant studies and reviews were identified through targeted searches in PubMed with search terms “sleep” AND (“inpatient” OR “hospital”), and supplementary screening of reference lists. The included literature was organized into five broad thematic domains that emerged during our narrative synthesis, and therefore reflecting the most investigated aspects: (1) environmental factors, (2) hospital infrastructure and room configuration, (3) patient-related factors, (4) care routines, and (5) interventions aimed at improving inpatient sleep.

## Thematic Findings

### Environmental Factors

#### Noise and Sound Levels

Noise consistently emerges as the most prominent environmental factor disrupting sleep in both general ward and intensive care settings [10–12]. Common sources of disruptive noise include nighttime staff activity, alarms, and sounds from medical equipment and neighboring patients - many of which are difficult for individual clinicians to control without deliberate institutional coordination.

Noise levels in patient rooms and ICUs frequently exceed WHO recommendations for sleep (> 35 dB), often with frequent high level (80dB) peaks [13, 14]. Shared patient rooms were found to have significantly higher ambient and peak noise levels compared to single occupancy rooms, highlighting the role of hospital design in mitigating noise disruption and its consequences for sleep quality [15]. These objective measurements are important in contextualizing patient-reported disturbance: they confirm that noise levels in many hospital environments are not merely subjectively unpleasant but exceed thresholds at which sleep fragmentation is known to occur.

#### Artificial Light Exposure

Adequate circadian regulation requires light during the day and darkness at night, conditions that hospital environments frequently fail to provide. Circadian disruption in hospitalized patients is therefore bidirectional: insufficient daylight during the day prevents normal entrainment of the sleep-wake cycle, while elevated artificial light at night further delays and fragments sleep [16]. These problems have been documented across settings; in pediatric inpatients for instance, insufficient daytime light and excessive light exposure at night were recorded simultaneously within the same patients [14], and light profiles in ICUs rarely align with natural day-night cycles [13]. The combined effect is a sustained disruption to circadian timing that may impair not only sleep architecture but also broader recovery processes dependent on circadian regulation.

Vulnerability to light-related disruption varies by patient characteristics and clinical context. Among respiratory inpatients on mixed wards, younger patients and those in shared rooms were most likely to report disturbance from nighttime lighting, while older patients appeared comparatively unaffected [10]. The authors speculated that this could be a consequence of age-related reductions in light sensitivity and subsequent effects on melatonin suppression, but the true underlying cause remains an open question.

Disruptive light conditions, both insufficient daylight during the day and excessive artificial light at night, can persist even in purpose-built modern facilities. For example, in an ICU that included large windows and modern lighting, constructed in 2016 in an area with many days of annual sunlight (San Diego, CA, USA), daytime bedside light levels remained below 150 lx [17]. These light levels are comparable to those in older buildings [17]. Window orientation influenced light intensity, but not to a clinically meaningful degree, underscoring that architectural investment alone is insufficient without deliberate lighting design. At the institutional level, fewer than half of top-ranked U.S. hospitals reported any active measures to reduce nighttime artificial light [18], indicating that awareness of the problem has not yet translated into systematic practice [17, 18].

#### Temperature and Other Physical Factors

Temperature has received comparatively little research attention as a sleep disruptor in hospital settings, though the available evidence suggests its impact may be underestimated. One study performed in a neurology inpatient unit found that temperature discomfort was significantly associated with lower patient-reported sleep efficiency [19]. Patients who identified temperature as disruptive had significantly lower sleep efficiency (73.9% vs. 80.4%, *P* = .04), though this association did not survive adjustment for clinical covariates (*P* = .24). This may suggest that temperature discomfort may partly function as a proxy for broader illness burden rather than an independent predictor of poor sleep. These findings illustrate the methodological challenge of isolating individual environmental contributors in complex clinical populations; nevertheless, the unadjusted association and the notable difference in sleep efficiency between affected and unaffected patients, taken together with emerging evidence of independent effects of environmental exposures (e.g. noise, temperature, air pollution) in the home [20], suggest that temperature warrants closer monitoring as part of routine environmental assessment in hospitals.

Beyond physical stimuli, the psychological novelty of the hospital setting itself is a recognized contributor to poor sleep. Sleeping in an unfamiliar environment - with unknown sounds, care routines, and reduced personal control - elevates arousal and impairs both sleep onset and maintenance, effects that are most pronounced in the early days of admission [21, 22]. This is analogous to the well-documented first-night effect observed in sleep laboratory research, whereby participants sleeping in an unfamiliar laboratory setting for the first time show poorer sleep quality, with disturbances largely resolving by the second night. By analogy, sleep disruption in hospitals may attenuate as patients habituate to ward conditions; however, for patients with short hospital stays, environmental novelty may remain an unresolved barrier to sleep throughout their entire admission.

### Hospital Infrastructure and Room Configuration

#### Room Occupancy: Multi-Bed vs. Private Rooms

Room occupancy is one of the most consistently reported structural determinants of inpatient sleep quality, with shared accommodation linked to poorer outcomes through several converging mechanisms. These include increased ambient noise, reduced privacy, and exposure to co-patients care interactions - including nighttime procedures, visitor activity, and equipment alarms - as well as potential mismatches in sleep-wake schedules between roommates [12, 23, 24]. The effect sizes of these shared accommodations can be substantial, for example one study found that 51% of patients in shared rooms reported poor or very poor sleep compared to 17% in single rooms [15]. This difference is likely due, at least in large part, to elevated noise levels in multi-occupancy room, with noise measurements showing maximum noise levels could exceed 100 dB L_AF, max_, far above thresholds compatible with restful sleep [15]. But beyond noise level alone, acoustical character of the sounds (e.g. frequency spectrum) and the context in which they occur (e.g. with accompanying caregiving and the addition of other environmental factors like light) is also highly important [25]. Overall, findings suggest that room configuration is not merely a comfort consideration but a physical determinant of patient sleep disruption, with direct implications for hospital design.

#### Level of Care and Ward Type

The level of care a patient receives also shapes sleep independently of room configuration. Patients in the ICUs consistently report the poorest sleep of any inpatient group, attributable in part to the density of medical devices and to disorientation arising from continuous care [5]. The effects can persist beyond the ICU stay. For instance, one study using an EEG-based metric of sleep depth and stability found that nearly half of critically ill patients had abnormal sleep architecture - minimal deep sleep or sustained hyperarousal - persisting in patients six months post-discharge [26].

### Patient-Related Factors

#### Pain

Pain is repeatedly identified as one of the most significant drivers of inpatient sleep disturbance, operating through a bidirectional relationship in which inadequate sleep heightens pain perception while pain itself disrupts sleep continuity, creating a self-reinforcing cycle [27]. Breaking this cycle requires that pain management be treated as an integral component of sleep-promoting care rather than a separate clinical concern, alongside attention to other physiological stressors such as discomfort from illness or medical devices.

Several studies, particularly in surgical and oncology populations, have demonstrated the negative impact of pain on sleep quality [28–30]. In women undergoing autologous breast reconstruction, sleep quality declined during hospitalization and remained impaired two weeks postoperatively, with 83% reporting poor sleep; those with moderate-to-severe pain (> 4/10) consistently reported worse sleep than those with lower pain [31]. This relationship extends across diagnostic categories: pain has been identified as a significant predictor of sleep disturbance in orthopedic surgery patients, paediatric inpatients, and hospitalized cancer patients alike [12, 32, 33], suggesting that inadequate pain management carries sleep-related consequences that are not limited to traditionally pain-focused settings.

The mechanisms by which pain disrupts sleep nonetheless differ across settings. In surgical patients, postoperative pain combines with anaesthetic recovery, immobility, and wound discomfort to impair sleep, while preoperative sleep quality predicts postoperative pain - again illustrating the bidirectional pain-sleep relationship [34]. In oncology, chemotherapy-induced nausea, fatigue, and metabolic disturbance compound pain [35], with the psychological burden of diagnosis adding anxiety and rumination. This combined physiological and psychological load may explain why oncology patients report among the poorest sleep of any inpatient group, and it implies that any systematic effort to protect inpatient sleep must treat pain management as a core component rather than a parallel concern.

#### Illness Severity and Anxiety

Patient anxiety is one of the most consistently identified psychological contributors to inpatient sleep disturbance, with validated instruments such as the Hospital Anxiety and Depression Scale showing that higher anxiety scores are associated with shorter sleep duration and poorer subjective sleep quality across diverse patient populations [29, 36, 37]. The consequences of anxiety extend beyond sleep: anxiety and depression have also been linked to heightened pain perception, cognitive dysfunction, and immune suppression, suggesting that anxiety functions as a node connecting sleep disruption to a broader cluster of adverse hospitalization outcomes [27]. Addressing anxiety during hospitalization may therefore yield benefits extending well beyond sleep itself.

Beyond environmental and care-related factors, the severity of underlying illness independently shapes sleep quality in hospitalized patients. Patients with a high disease burden, such as those with cancer, frequently experience short sleep duration and low sleep quality [38]. A cross-sectional study of 246 cancer inpatients in Vietnam, primary late stage and being treated with chemotherapy, found 58.5% had poor subjective sleep (mean PSQI 7.5; average duration 5.4 h), with effects especially pronounced among those with depression [35]. The effects of disease severity and accompanying depression may be especially pronounced among older adults, who often have preexisting disruptions in sleep regulation and heightened sensitivity to the physiological and psychological stressors of hospitalization [39, 40].

Several mechanisms plausibly link high disease burden to poor sleep. The most direct is symptom load: pain, dyspnea, nausea, pruritus and treatment side-effects fragment sleep and prolong sleep onset [27, 35]. Greater disease burden further entails greater care intensity, more frequent nocturnal observations, infusions and medication rounds, alongside immobility that reduces daytime activity and daylight exposure, weakening circadian entrainment [16]. Finally, the psychological burden of advanced illness, including anxiety, depressive symptoms and existential concern, sustains cognitive and physiological arousal at night, consistent with the pronounced effect observed among patients with concurrent depression [27, 35]. These pathways are unlikely to act independently, and the cross-sectional designs of the available studies cannot separate them.

#### Sleep in Psychiatric Inpatients

In psychiatric settings, sleep disturbance is entwined with core symptoms rather than being an environmental side effect: impaired sleep both drives and is driven by mood instability and emotional dysregulation, independent of the physical environment [41]. Sleep here is also markedly fragmented, with poor nights leading to daytime napping that further undermines circadian regulation [42]. These dynamics carry particular clinical weight; poor sleep quality is linked to increased suicide risk and worse outcomes after admission [43–45], positioning sleep as both a predictor and a modifiable target in psychiatric care.

#### Sleep in Paediatric Inpatients

Pediatric wards present a distinct sleep disruption profile, shaped less by the ward environment than by separation from home routines and parental presence. One study examined parent-reported sleep disruption in pediatric ICU (PICU) patients and compared it to sleep in a hospital-based pediatric sleep laboratory [46]. It found that 71% of PICU children experienced significant disruption, most commonly from pain (38%), being away from home (30%), and alarms (28%), while light was rarely implicated (5%) - contrasting with adult ICU studies. PICU children slept worse than children in a hospital sleep lab, suggesting that critical illness and separation from home routines matter more than the hospital environment alone.

### Care Routines

Nighttime patient-staff interactions (PSIs) consistently emerged as a key disruptor of inpatient sleep. A retrospective study of overnight vital sign monitoring in 354 stable pediatric surgery patients found that although 62% had at least one abnormal reading and 58% prompted an intervention (usually medication), serious outcomes were rare (ICU transfers 1.4%, unplanned surgery 0.9%) - suggesting routine nighttime monitoring offers limited clinical utility relative to its impact on sleep [47].

Another study examined the impact of nighttime PSIs on sleep opportunities among neurology inpatients, using electronic health record data from over 3,300 patient nights [48]. Sleep disruption was quantified using surrogate measures such as Longest Uninterrupted Sleep Opportunity (LUSO) and interruptive episodes. PSIs occurred on 92% of nights, with an average of 2.81 interruptions and 4.06 h of LUSO per night. While vital signs and medication administration were the most frequent PSIs, the greatest reductions in sleep opportunity were caused by neurological checks and off-unit diagnostic testing, each reducing LUSO by approximately 40 min. Taken together, these findings challenge the assumption that routine nighttime monitoring is uniformly necessary, and point toward the conclusion that consolidating, deferring, or eliminating lower-priority nighttime interactions would preserve sleep without compromising clinical safety in stable patients.

### Interventions

#### Pharmacological

A 2026 network meta-analysis provided a comprehensive comparative framework for pharmacological management of inpatient insomnia, synthesizing 29 RCTs across 1,913 patients and seven drug classes including benzodiazepines, Z-drugs, melatonin receptor agonists, antidepressants, antihistamines, alpha-2 adrenergic agonists, and dietary supplements [49]. The findings offer important context for interpreting the individual drug class evidence reviewed below, and highlight a critical gap: geriatric and critically ill inpatients - the populations most vulnerable to both poor sleep and pharmacological adverse effects - remain substantially underrepresented in the trial literature, with only six of 29 studies enrolling geriatric patients and only two conducted in intensive care settings.

##### Pharmacological Pain Management

Among analgesic strategies, esketamine has attracted the most concentrated recent research on sleep-specific outcomes, owing to its NMDA-receptor antagonism, which may confer sleep-modulating properties beyond analgesia [50, 51]. Other approaches such as multimodal analgesia influence sleep only indirectly and have received little recent attention, making esketamine the primary pharmacological focus here. The evidence for a direct sleep benefit appears to depend heavily on surgical context and comparator: in hip replacement patients, esketamine improved pain control but did not outperform fentanyl on sleep quality, suggesting that analgesic benefit alone may be insufficient to overcome other drivers of postoperative sleep disturbance [52]. By contrast, in gynecological and thoracic surgery populations, esketamine reduced postoperative sleep disturbance more effectively than standard care and outperformed dexmedetomidine, with benefits persisting up to two weeks postoperatively [53, 54]. Together, these findings suggest esketamine’s sleep-promoting effects may be most pronounced when pain is a dominant driver of disturbance, though the overall evidence base remains preliminary and warrants confirmation in larger trials [55].

##### Melatonin

Melatonin is a chronobiotic - it can shift sleep timing but has no established efficacy for reducing sleep onset latency or increasing total sleep time - yet it has become the most frequently prescribed agent for inpatient sleep complaints, particularly new-onset insomnia [56]. Large-scale studies confirm this for delirium prevention: melatonin receptor agonists showed no advantage over zolpidem or temazepam in a cohort of over 22,000 surgical patients, and an RCT found no difference in delirium severity versus placebo [57, 58]. This conclusion is now supported at the comparative level by a 2026 network meta-analysis, which found that melatonin receptor agonists failed to demonstrate significant improvement on any of the four measured sleep outcomes - subjective sleep quality, sleep onset latency, total sleep duration, and nocturnal awakenings - compared to placebo [49]. This held across adult and geriatric populations, reinforcing that melatonin’s use as a hypnotic is unsupported regardless of age.

Nevertheless, melatonin prescribing remains frequent: one study reported that 28% of 754 inpatients received a sleep medication (mostly melatonin), yet only 16% had a documented sleep assessment and 3% any nonpharmacological intervention [59], and melatonin administration in Danish ICUs more than doubled between 2015 and 2019 despite the weak evidence [60]. These findings highlight a disconnect between melatonin’s identity as a phase-shifter and its application as a sedative-hypnotic - a pattern that risks perpetuating misinformation and delaying adoption of better-supported nonpharmacological approaches.

On the other hand, ICUs may be an exception. Critically ill patients show markedly reduced endogenous melatonin secretion, so exogenous melatonin here may restore depleted chronobiotic signaling rather than act as a hypnotic. A 2025 meta-analysis of 32 RCTs (3,895 patients) found low-certainty evidence that melatonin may reduce delirium (RR 0.72), slightly shorten ICU stay, and modestly improve perceived sleep [61]. This does not alter melatonin’s lack of hypnotic evidence in general wards, but flags the ICU as a context where its circadian role warrants further study.

##### Z-Drugs and Benzodiazepines

Benzodiazepines and Z-drugs are frequently prescribed for inpatient sleep despite safety concerns that are particularly acute in older adults. Age-related declines in hepatic and renal clearance markedly prolong half-lives - nitrazepam from ~ 29 to ~ 40 h, lorazepam from 8 to 25 h - causing drug accumulation and elevated effective concentrations [49]. These pharmacokinetic changes increase the clinical risk of residual sedation, falls, cognitive impairment, and delirium in this population, and are compounded by heightened central nervous system sensitivity with aging. Despite these concerns, short-acting benzodiazepines - specifically triazolam and brotizolam - may be relatively safe for short-term hospital use, with significant reductions in sleep onset latency and nocturnal awakenings over a median of seven nights even in geriatric populations, provided administration is time-limited and doses are appropriate for age and frailty [49]. By contrast, Z-drugs do not demonstrate significant superiority over placebo despite positive findings in individual trials, suggesting their widespread use may not be justified by the comparative evidence [49]. Despite this, prescribing remains high: 15% of over 21,000 surgical inpatients received postoperative sleep medication, most commonly benzodiazepines [62], and a structured intervention was needed to cut benzodiazepine prescribing from 24.3% to 8.5% in older inpatients - showing that organizational measures can address inappropriate use [63].

##### Antipsychotics and Antidepressants

Antipsychotics (e.g., quetiapine) and antidepressants (e.g., trazodone) are occasionally used for inpatient sleep disturbances, however, the limited evidence base and potential for clinically meaningful adverse effects mean neither antipsychotics nor antidepressants are currently supported as first-line pharmacological options for inpatient sleep disturbance [64]. Evidence from psychiatric inpatients further suggests that treating the underlying condition improves sleep more than hypnotics do: in one study, sleep duration improved more in patients admitted with mania (1.14 h) than in those admitted with major depression (0.37 h), despite lower hypnotic use in the manic group [65]. Shorter sleep duration at admission also predicted longer stays, underscoring that sleep is best regarded as an integral part of psychiatric treatment.

#### Nonpharmacological

Nonpharmacological interventions represent the most evidence-supported and clinically recommended approach to improving inpatient sleep (1), encompassing strategies that range from environmental modifications and care routine restructuring to physical, psychological, and sensory therapies. A 2026 meta-analysis synthesizing 36 studies across 16 intervention types for ICU patients identified music therapy as the intervention with the highest probability of being most effective (94.9%) [66]. This was followed by aromatherapy (76.2%), and with eye masks, combined eye mask and earplug use, and multicomponent interventions also demonstrating significant improvements over standard care [66]. These findings offer an important framework for prioritizing interventions in resource-constrained settings, and the mechanisms proposed by the meta-analysis authors - reduction of environmental disturbance alongside stabilization of autonomic and neurotransmitter activity – may help explain why several seemingly disparate intervention types converge on similar outcomes.

##### Staff Education and Nightly Checks

A substantial proportion of nighttime sleep disruption stems not from clinical necessity but from how routine care tasks are scheduled, and a growing body of evidence shows that restructuring these workflows can meaningfully protect sleep. Structural changes at the level of electronic health record order sets can eliminate unnecessary nighttime activities by default, embedding sleep-friendly practice into the workflow itself rather than relying on individual staff discretion [67]. Where implemented, effects are measurable: cancelling non-urgent overnight care in a PICU raised uninterrupted sleep from 32% to 49%, and postponing vital sign checks and medication added 40 min of sleep with fewer awakenings [68–70]. Bundling nighttime care reduced sleep aid use, falls, and costs while improving satisfaction [71]. Patients also preferred wearable monitors over manual checks, though the technology is not ready to replace them. Current devices are limited by motion artefact and false alarms, by incomplete capture of the parameters required for established early warning scores, and by uncertain accuracy in patients with arrhythmia or poor peripheral perfusion [72]. Despite this accumulating evidence, organizational change has lagged: 96% of top-ranked US hospitals acknowledge the importance of sleep, yet most lack the staff practices needed to act on this, pointing to a persistent gap between institutional awareness and operational culture [18]. A cluster RCT in general medical wards corroborates the value of restructuring morning care timing specifically: delaying early morning nursing rounds was among the components of a multicomponent bundle that produced significant sleep quality improvements, suggesting that the sleep-disruptive effects of care scheduling extend beyond the nighttime hours into early morning transitions [73].

##### Noise and Light Optimization

Environmental optimization shows benefits that vary by population: white noise improved sleep in premature infants [74], while in adults a noise-reduction project added only ~ 8 min per night [37], underscoring that noise control alone is insufficient amid multiple disruptors. For lighting, a Turkish ICU RCT found cyclic day–night lighting increased total sleep time by ~ 2.6 h versus standard lighting, with gains in deep and light sleep [75].

##### Sleep Bundles

Bundled interventions - which combine multiple sleep-promoting strategies into a single structured package - have shown promise both for directly improving sleep and for reducing inappropriate pharmacological prescribing. A surgical post-operative pack combining comfort items (earplugs, eye masks) with a sleep-protective order set produced substantial improvements in both sleep duration (6.4 vs. 4.7 h) and patient satisfaction, with 92% reporting they would use it again [76]. At the institutional level, a hospital-wide sedative-hypnotic reduction bundle incorporating order set changes, medication reviews, and staff education successfully cut new sleep medication prescriptions from 15.5% to 9.1% without increasing adverse events [77]. Finally, an RCT in elderly surgical ICU patients found that a multicomponent protocol combining environmental modifications with earplugs and eye masks reduced seven-day delirium incidence from 17.0% to 10.1% (and from 30.0% to 14.0% in a high-risk subgroup), despite no measurable change in subjective sleep quality [78].

Outside the ICU, ward-level bundle evidence is scarcer. In one study conducted across six medical departments in a Dutch academic hospital, a bundle of delayed morning rounds, staff training, optimized medication timing, earplugs and eye masks, and evening sleep rounds, significantly improved second-night sleep quality in 291 patients (RCSQ 66.6 vs. 55.7, *p* = .033), though sleep quantity, delirium, and 30-day mortality were unchanged [73]. Adherence ranged from 42% to 73%, reflecting real-world implementation challenges even in well-resourced settings. Taken together, the evidence for bundled interventions is promising but should be interpreted cautiously. Of the studies reviewed here, only this ward-based trial demonstrated an improvement in subjective sleep quality, and it was modest and short-lived [73]. Bundles may therefore confer clinical benefit, but current evidence does not establish a consistent effect on sleep quality across hospital settings, and adequately powered trials incorporating objective sleep measures are needed.

##### Eye Masks and Earplugs

Eye masks and earplugs represent some of the simplest and lowest-cost interventions evaluated for inpatient sleep, and both have been independently identified as significantly effective in the most recent comparative evidence, with combined use ranking among the top interventions across 16 types evaluated in a 2026 network meta-analysis [66]. Even at low usage rates of 12–16%, one study still observed increased sleep time, suggesting a meaningful effect even among only partially adherent patients [68]. In pediatric cardiac surgery patients, eye masks reduced postoperative pain alongside improving sleep [79], while in the ICU, combined use of eye masks and earplugs was associated with both better sleep quality and lower rates of delirium compared to non-use [80] - extending the relevance of these devices beyond subjective comfort to a measurable clinical outcome. In cardiac critical care patients, three nights of eye mask and earplug use increased endogenous melatonin, shortened sleep latency, and reduced awakenings [81], highlighting that blocking light and noise can preserve sleep and circadian phase disruptions even in critical care.

##### Massage and Physical Therapy

Touch-based and physical therapy interventions, including acupressure, massage, and progressive muscle relaxation, have demonstrated consistent benefits for inpatient sleep across markedly different populations. In cancer patients, auricular acupressure cut sleep disturbance by 45% while reducing fatigue, pain, and opioid use, and progressive muscle relaxation improved sleep during chemotherapy [82, 83]. Comparable benefits appear across the age spectrum - from premature infants (massage) to burn and stem-cell-transplant patients (acupressure, acupuncture) - improving sleep efficiency and reducing wake after sleep onset [74, 84, 85]. Their consistency across such heterogeneous contexts suggests a shared mechanism - likely reduced sympathetic arousal - warranting further study.

##### Mindfulness, Auditive Therapy, Breathing Exercises and Aroma Therapy

A diverse set of mind-body and sensory-based interventions - including mindfulness training, music and audio therapy, breathing exercises, and aromatherapy - have been evaluated for inpatient sleep, and a consistent pattern emerges across this heterogeneous evidence base. Nearly all of these approaches improve sleep alongside a reduction in anxiety, reinforcing the bidirectional anxiety-sleep relationship discussed earlier in this review. In laryngeal cancer patients, mindfulness-based stress reduction improved subjective sleep quality alongside reductions in anxiety and depression and gains in social functioning [86], and in patients recovering from myocardial infarction (MI), music therapy combined with cardiac rehabilitation outperformed rehabilitation alone by improving sleep quality [87]. Sensory interventions help even in high-acuity settings; audiobooks in the ICU improved sleep while lowering pulse and blood pressure [21], and breathing exercises improved sleep and anxiety in surgical patients [88, 89]. Aromatherapy with rose oil produced comparable improvements in sleep and anxiety in burn patients [90–92]. Collectively, these findings suggest that interventions targeting psychological arousal, regardless of modality, might represent an effective and low-risk avenue for improving inpatient sleep across diverse clinical populations. This is supported by a recent meta-analysis finding that music therapy and aromatherapy were the two most effective nonpharmacological interventions for ICU sleep across 16 evaluated types [66].

## Discussion

Sleep is a vital yet overlooked component of recovery, with disruption linked to delayed healing, immune impairment, prolonged stays, delirium, and higher costs [1, 3, 27]. This review highlights the multifactorial nature of sleep disruption in hospital settings, spanning environmental factors, care routines, pharmacological and non-pharmacological aids, reinforcing that no single strategy is sufficient to address it in isolation [1, 8]. Despite widespread acknowledgement of sleep’s importance for health, recent implementation of sleep-promoting strategies remains inconsistent across hospital settings [18]. Several factors explain this gap: workflows prioritize monitoring schedules over rest, generating interruptions of limited clinical necessity [47, 48]; cultural barriers and inadequate sleep-hygiene training slow change [3, 18]; few hospitals conduct systematic sleep assessments, so poor sleep goes unaddressed [59]; and heterogeneous evidence across populations and wards impedes standardization [1, 8]. Recent network meta-analyses are beginning to address this gap by providing comparative rankings across different intervention types [66], though replication in non-ICU settings and with objective sleep measures remains needed. Furthermore, no formal policy or institutional incentives currently exist in the US to promote inpatient sleep, though awareness is rising [18], and most intervention studies remain small or short-term, limiting confidence in effect sizes and long-term sustainability.

Despite these limitations, the evidence supports actionable conclusions. Both system-level interventions (workflow restructuring, environmental redesign) and low-cost measures (earplugs, sleep masks, physical therapy) improve sleep-related outcomes including delirium, pain, and length of stay [1, 8, 71, 80], with one RCT showing that simple environmental protocols reduced delirium in high-risk ICU patients [78]. Specialized environments - ICUs, pediatric, and psychiatric wards - present distinct challenges requiring tailored rather than generalized approaches [5, 42, 46].

## Conclusions

Hospitals that treat sleep as a core clinical priority rather than a comfort concern are better placed to embed evidence-based interventions into standard care [27]. This will require further research on the long-term impact of sleep-focused care to convince leaders that such investments yield clinical returns [1, 8], alongside systematic sleep assessment, workflow redesign, and staff education to shift clinical culture [18, 48, 59] - together offering a scalable path toward treating restorative sleep as fundamental to recovery.

## Key References

- Beswick AD, Wylde V, Bertram W, Whale K. The effectiveness of non-pharmacological sleep interventions for improving inpatient sleep in hospital: A systematic review and meta-analysis. Sleep Med. 2023;107:243 − 67.
  - ○ This meta-analysis provides much background the interventions section, summarizing evidence on earplugs, light reduction, and staff routines. It also establishes the evidence base for non-drug strategies.
- Hurley-Wallace AL, Bertram W, Johnson E, Wylde V, Whale K. An opportunity to sleep well in hospital: development of a multi-level intervention to improve inpatient sleep (ASLEEP) using behaviour change theories. BMC Psychol. 2024;12(1):788.
  - ○ This paper describes ASLEEP, a program aimed at helping hospital patients sleep better. Using behaviour change theory and input from both staff and patients, they created practical strategies to improve ward routines, education, and the sleep environment. This is a key behavioural framework paper for multi-level hospital sleep improvement.
- Beltrami FG, Nguyen XL, Pichereau C, Maury E, Fleury B, Fagondes S. Sleep in the intensive care unit. J Bras Pneumol. 2015;41(6):539 − 46.
  - ○ This paper reviews the literature and summarizes how sleep is affected negatively in the ICU-setting compared to normal sleep. Furthermore, it states the implications for inpatients exposed to poor sleep.
- Koshy K, Gibney M, O’Driscoll DM, Ogeil RP, Young AC. Factors affecting sleep quality in hospitalised patients. Sleep Breath. 2024;28(6):2737-40.
  - ○ This paper reveals the poor sleep inpatients are exposed to in a ward setting compared to their home setting. Also identifies factors contributing to the disturbed sleep in the ward.
- Affini MI, Arora VM, Gulati J, Mason N, Klein A, Cho HJ, et al. Defining existing practices to support the sleep of hospitalized patients: A mixed-methods study of top-ranked hospitals. J Hosp Med. 2022;17(8):633-8.
  - ○ This study provides real-world data on how hospitals address or neglect inpatient sleep.

## Data Availability

No datasets were generated or analyzed during the current study.
